# A review on sliding wear properties of sustainable biocomposites: Classifications, fabrication and discussions

**DOI:** 10.1016/j.heliyon.2023.e14381

**Published:** 2023-03-08

**Authors:** Harikrishnan Pulikkalparambil, Ajish Babu, Anusree Thilak, N.P. Vighnesh, Sanjay Mavinkere Rangappa, Suchart Siengchin

**Affiliations:** a Natural Composites Research Group Lab, Department of Materials and Production Engineering, The Sirindhorn International Thai-German Graduate School of Engineering (TGGS), King Mongkut's University of Technology North Bangkok, Bangkok 10800, Thailand; b Department of Metallurgical and Materials Engineering, Indian Institute of Technology Patna, Bihta, Patna-801106, India; c Department of Polymer Science and Rubber Technology, Cochin University of Science and Technology (CUSAT), Kochi, Kerala 682022, India

**Keywords:** Sliding wear, Tribology, Biopolymers, Composites, Biofillers, Synthetic fillers

## Abstract

Biocomposites have gained huge attention in the field of manufacturing. They are widely accepted over conventional petroleum-based composites due to less environmental footprint and safer living habitats, abundance, availability, recyclability, reusability, and end-life disposals. The potential applications of biocomposites are now widely accepted in key engineering areas such as automotive, construction, consumer products, and aerospace industries. Concurrently, tribological properties for biopolymer composites are an appealing research direction. In this review article, a comprehensive literature survey of recent progress made in sliding wear properties of biocomposites are discussed in detail. It summarizes natural and synthetic ways to attain tribological performances in biocomposites such as biopolymers with bio-fillers, biopolymers with synthetic/inorganic fillers, and non-biopolymers with bio-fillers. The study gives a deeper understanding of the crucial informations regarding sliding wear properties of biocomposites and thereby aid in the future research in the design and preparation of similar composites.

## 1 Introduction

**Fig. 1:**
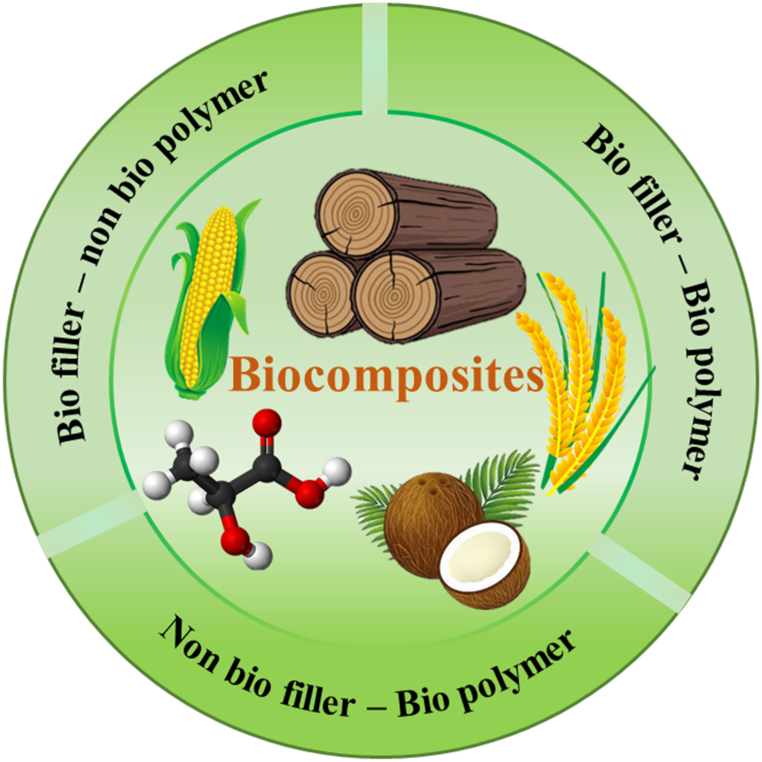
Illustration of types of biocomposites.

Polymer composites have previously demonstrated their significance as lightweight materials for the replacement of heavy metal structures [1]. These are widely employed in the structural and vehicle industries because they give excellent mechanical qualities and high-quality designed products. Many efforts have still been made to develop composites with excellent performance and multifunctionality. Apart from the well-established traditional composites having macro reinforcements, nanocomposites have also gained great attention because of their extraordinary properties [2]. Such composites are well used in electronic, biomedical, marine, aerospace, and infrastructure applications [[3], [4], [5], [6], [7], [8], [9]]. However, the disposal of polymer composites, primarily composed of synthetic/inorganic materials has become a significant concern for many sectors [10]. With growing environmental concerns and the depletion of non-renewable sources, the interest in products based on renewable sources is increasing [11]. As a part of this global transition toward sustainability, the use of conventional polymers for the fabrication of composites has become a severe challenge for many industries [10]. In this vein, eco-friendly biocomposites prepared from natural resources have been extensively developed and researched in recent decades [12,13]. Biocomposites consist of one or more phases that have a bio-origin [14]. It can be of both reinforcement and polymer matrix derived from a biosource, or any of the phases from biosources, as illustrated in Fig. 1. As reinforcement in the polymer matrix, bio-based fibers, agricultural waste, seed powders, animal waste, and another biowaste, are used to fabricate biocomposites [15]. In matrix phases, biodegradable polymers such as polylactic acid, starch, chitosan, hydroxypropyl methylcellulose, and polyvinyl alcohol, have been used with natural fiber to produce biocomposites [16,17]. Benefits such as less environmental footprint and safer living habitats, lightweight, abundance availability, recyclability, reusability, and end-life disposal potential make them more attractive than conventional composites. However, the most challenging aspect of biocomposites is acquiring the desired properties for specific applications. A wide range of biobased materials have been developed to improve the chemical, morphological and mechanical qualities of polymer composites, and yet efforts are ongoing to achieve better properties [18].

According to reports, the wear of materials accounts for 63% of total costs in industries. Furthermore, elements like wear, friction, and heat cannot be eliminated; they can be mitigated [19]. In general, polymer composites used in brake pads, rotating parts, pressure tanks, bushings, tank liners, etc., are less stable and prone to failure, making achieving the needed property difficult. In automotive, the wearing of composites is a disadvantage, metals are generally used in sliding components but due to lightweight requirements, they are switched over to polymer composites [20]. As discussed earlier, owing to the shift towards biobased materials, the role of biocomposites for wear applications has also been discussed by many researchers [21,22]. Certain fillers and polymers with their self-lubricating characteristics have been identified in the reduction of wearing in polymer composites. However, a general comparison of the wear behavior of biocomposites with non-fibrous naturally sourced fillers is not yet reviewed. In this chapter, the sliding wear behavior of biocomposites fabricated using fillers other than natural fibers, against polished metallic counterparts is discussed.

## 2 Sliding wear mechanism and methods

Wear is described as the loss of matter from the surface of material caused by movement, resulting in a reduction of mechanical characteristics. According to the ASM/TSS Thermal Spray Terminology and Company Origins document, sliding wear is defined as “The motion of two moving bodies in which these surface velocities, at the point of contact, are different about magnitude and/or direction”. Wear is a property of a material that is determined by the environment, the surface's contact area, the material's microstructure, sliding motion, and the topological qualities of the surface. As a result, wear is more accurately described as material wear [23].

**Fig. 2:**
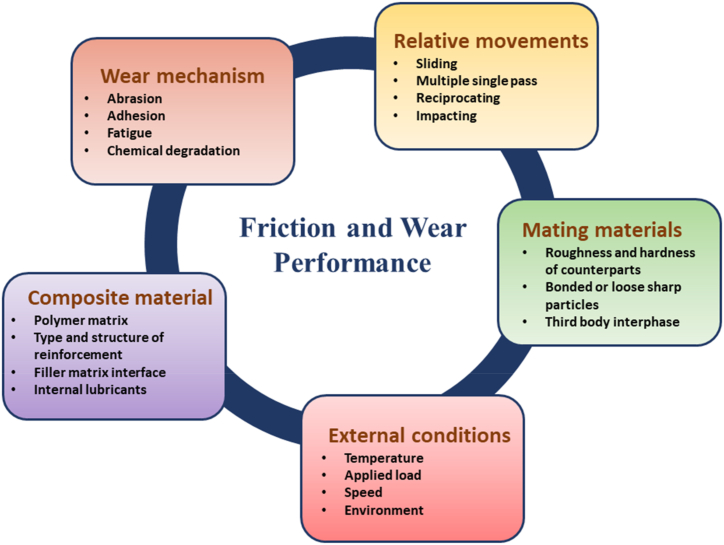
Illustration of various factors affecting the frictional and wear performance of polymer composite [24].

Polymer composites as emerging functional materials due to their wide tailoring opportunities and peculiar properties have been extensively used in the automotive industry. Their uses as a structural component for tribological loading applications have increased over the past few decades. One should note that, the wear of polymers and their composites is not a simple material's property. As shown in Fig. 2, it highly depends upon the wear conditions, wear mechanisms, materials type, surface behavior, and the environment [24]. For instance, the polymer composite that shows a good wear resistance against polished metal counterparts should not essentially be able to perform against an abrasive rough surface. So, understanding the application requirement for optimizing the composite properties is important.

## 3 Classifications of wear in biocomposites

Wear is classified mainly into 6 types such as abrasive wear, erosive wear, adhesive wear, surface fatigue, corrosive wear, and fretting wear [25]. The understanding of wear behavior depends upon the application and testing methods. For instance, for a composite part that is undergoing frictional conditions without any harsh environment, sliding or abrasive wear tests are enough to understand the wear resistance of the material. Generally, for polymer composites, where the wear behavior for most of the applications doesn't need any harsh environments, sliding wear tests are exhibited; dry sliding wear and wet sliding wear. In dry sliding, when a polymer composite slides over the counter face surface, the sliding force becomes more than the cohesive force acting between the polymer molecules themselves. This results in a transfer layer formation on the counterface [26]. The formation of the transfer layer and its bonding with the polymer and sliding surface has high significance to determine the wear property of the polymer. The influence of fillers in composites also plays a vital role in stabilizing and interlocking the transfer layer, thus determining the wear property [26]. Moreover, the wear rate is influenced by the sliding speed, normal load, and sliding distance when repeatedly sliding over a metal surface. The wear rate in polymer composites rises with the reduction of sliding distance, and increases in applied load and sliding speed [27]. On the other hand, in wet sliding, liquid facilitates the formation of a layer between polymer and metal surfaces, therefore it can reduce the coefficient friction between both of them. As a result of the reduced wear, water-absorbing brake pads in automobiles can be produced to improve wear [28]. Several testing methods are adapted for rating the wear performance of polymer composites such as tribometer testing, dry sand rubber wheel, block on disk, block on the ring, pin on the drum, and pin on disc [25,29,30]. For sliding wear behavior of composites, pin on disc method with steel or metal counterparts are commonly used.

## 4 Wear behavior of elastomer, thermoset & thermoplastic

Polymer composites are widely employed in automotive sectors due to their low weight and lubricating characteristics. Polymer composites can benefit from the inclusion of appropriate fillers to improve their sliding wear [31]. During the application of frictional force, the composites containing fibrous reinforcing fillers such as aramid, glass, and carbon fibers could exhibit heat generation, which can lead to loss of mechanical properties [32]. The commonly used thermoplastics are polypropylene, polyamide, polystyrene, high-impact polystyrene, polyethylene, and polyetheretherketone. While phenol-formaldehyde resin, polyesters, and epoxy resins, are commonly utilized in thermosets for wear application. Elastomers include natural rubbers, polybutadiene, polyisoprene, chloroprene, ethylene-propylene rubber, butyl rubber, silicone rubbers, etc. [33].

Wearing in elastomers is determined by sliding velocity and slip; a higher slip velocity results in more material being worn [34]. The continuous sliding of elastomeric composites induces specific compression-strain cycles which form wrinkles above the rubber surfaces, triggering fracture propagation, known as Schallamach's wave [35]. When elastomers are subjected to slide against a fresh counterpart, frictional forces increases. However, on repeatedly sliding, due to the rubber deposits on counterparts, a decrease in frictional forces, thus enhancement in wear property are observed. On the other hand, during sliding, free radicals from rubber segments can form metal oxide-polymer complexes and cause wear of metallic counterparts too [36]. Addition of fillers in rubber that can form layers on the metal surface are adopted to reduce wear resistance in elastomeric composites [37].

The wear rate of thermoplastic composites has a considerable link with their mechanical characteristics [38]. In thermoplastic composites reinforced with natural fibers, the natural filler has a greater impact on enhancing wear resistance. Studies have shown that wood polypropylene composites have a 40% increase in wear resistance. These advancements are game-changers in the field of sustainable materials, offering improved tribological qualities [39]. Elastic fatigue causes wear in thermosets, which can be minimized by incorporating elastomers into pure thermosets.

The wear behavior of thermoset composites has some modest instabilities in the high-temperature zone. In composites, the wearing pattern is largely determined by the orientation of the fibers; maximal wear may be found in perpendicular orientations, whereas least wear can be found at 45° [40]. Wearing is greatly reduced in high-performance polymers due to their low cohesive energy, which is a product of microstructure and functionalities [41]. When compared to polyester, the epoxy-based matrix has superior wear resistance [42].

## 5 Fabrication techniques of biocomposite

**Fig. 3:**
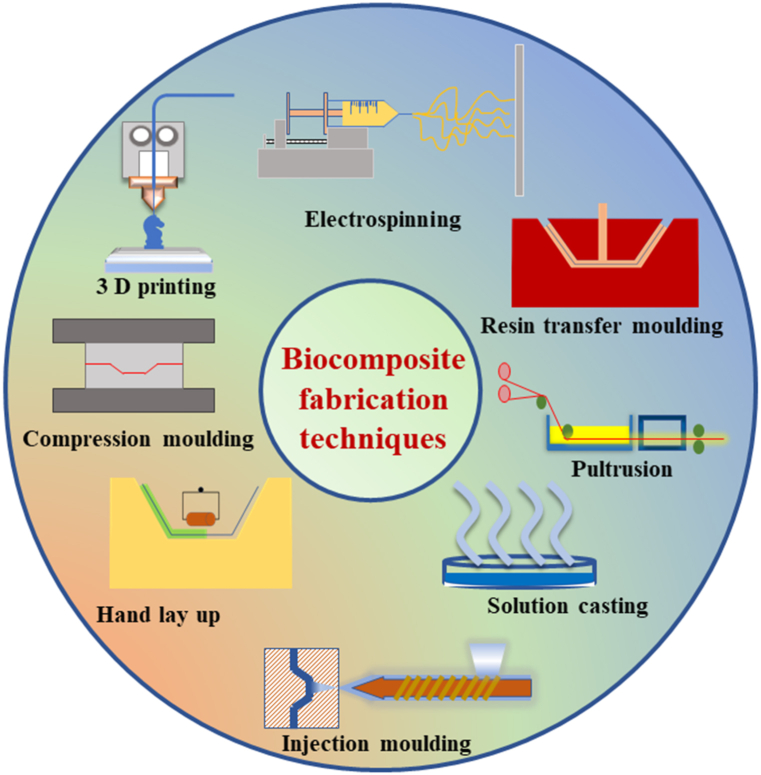
Illustration of different techniques used for biocomposite fabrication.

Green biocomposites are prepared by a variety of processing methods. Owing to their, acoustic, mechanical, and morphing capabilities, as well as their low density, decreased environmental imprint, and better end-of-life management, bio-derived fillers are employed as composite reinforcement to replace synthetic fillers [43]. However, they cannot be employed as a composite reinforcement in the same manner as synthetic equivalents may. To pick them appropriately, a thorough understanding of their microstructure and composition-related features, which vary from one type of bio filler to another, is vital. It is also crucial to analyze their susceptibility to heat and humidity, intricate polysaccharide composition, intrinsic flaws, and natural variability to achieve good performance [44]. The dimensions, shape complexity, mechanical qualities, and performance of composite materials might vary depending on the processing technique and context [45]. Additionally, processing parameters such as temperature, pressure, and time are also critical in fabricating the biocomposite [45]. Fig. 3 illustrates the different methods used for fabricating biocomposites. As one can see that the methods used to prepare biocomposites are mostly analogous to those used to develop plastics or composite materials.

Considering the cost-effectiveness and simplicity, methods like hand lay up, spray lay up are widely applied for the fabrication of biocomposites [46,47]. Although these methods have advantages of the easy procedure, and usage of various fiber and matrix combinations, the lack of control over resin viscosity, and labor competence limit their use across applications. Other conventional techniques for manufacturing biocomposites are compression molding and resin transfer molding. This method has been favored by many researchers for the fabrication of biocomposites [48,49]. However, the slowness of these conventional techniques impacts manufacturing timelines when large volumes are required. Furthermore, the effect of pressure, temperature, and volume fraction of filler and matrix impact significantly the properties of composites [50]. For the high-volume manufacturing of biocomposites, the injection molding process is commonly utilized. The typical injection molding process comprises feeding material in pellet form through a hopper. For natural fiber composite pellets, a hopper drier is recommended to ensure the pellets stay dry during the injection process [51]. When compared to compression molded products, conventional injection moldings would be inferior regarding the mechanical performance, since the former could only hold short reinforcing fibers having low aspect ratios, whilst the latter can accommodate long or continuous fibers. Apart from conventional injection molding, other process variants such as micro-injection molding for micro and components [52], high-speed injection molding of ultrathin composite parts [53], and injection molding of long-fiber-reinforced thermoplastics [54], that exhibits excellent mechanical properties for composites have also been developed.

Pultrusion is a composite fabrication technology that involves impregnating continuous fibers with a polymer matrix through a heated die to produce biocomposites [55,56]. It can run continuously to make components with ceaseless cross-sectional profiles on a massive scale, which is challenging to attain using other composite production techniques. Researchers have used the pultrusion technique to manufacture the continuous composite using flax fiber reinforced polypropylene to understand the mechanical performance of composites [56]. Advanced processing techniques, such as the combination of pultrusion and reaction injection molding (RIM) have also been investigated for the development of the RIM pultrusion (reactive pultrusion) process [57]. Axisymmetric components like pipes, tubes, driveshafts, and pressure vessels are manufactured by a filament winding process [58]. This technique is ideally suited for axisymmetric biocomposite products as it has certain advantages over other manufacturing techniques, such as the ability to generate components with high fiber volume fraction (60–80%) and high specific strength. Furthermore, this process has also been utilized to fabricate composites for biomedical applications [59,60]. Another simple method for biocomposite fabrication is solution casting. It is usually a low-temperature process and provides films with uniform thickness, optical purity, low haze, and isotropy [61]. Based on the interaction of specified fillers and polymers, both aqueous and non-aqueous solvents are utilized. Even though preparing nano reinforcement-based biocomposites is simply done through solvent casting, the sluggish processing speed, harmful solvent usage, and expense for solvent recovery limit the application of this approach to the laboratory scale [62].

Electro-hydrodynamic techniques like electrospinning have been extensively researched in the biomedical field over the last decade, owing to the obtained fibers' submicrometric diameter, and consequently, the large surface area to volume ratio and high porosity with interconnected voids formed between the fiber structures [63]. Researchers have developed nanocomposite employing the incorporation of nanoparticles in a polymer solution, where the resulting materials consist of nanoparticles-embedded polymeric fibers with enhanced properties [64].

Moreover, due to the spinnability of natural and biodegradable polymers, a tandem procedure, involving simultaneous electrospinning of a polymer solution and electrospraying of nanoparticles dispersion to attain nanoparticles-coated polymeric fibers for drug delivery and tissue engineering applications [65]. Electrospinning was identified as the most promising fabrication method for mimicking collagen nanofibers of extracellular matrix by biodegradable biopolymers or biocomposites as a replacement for injured tissue [66]. Although electrospinning is a low-cost, simple technology for forming fibrous structures, the difficulties in obtaining enough fibers to create large-scale designed structures in a reasonable time frame restrict its applications. As a result, electrospun fiber-reinforced materials are generally limited to medical applications such as wound dressings, medical implants, and tissue regeneration scaffolds.

Additive manufacturing (AM) refers to a class of technological developments that have seen tremendous growth over the past decade. These processes are categorized based on the nature of the material, which can be solid, liquid, or powder-based, and how it is selectively deposited, solidified, or glued together layer by layer to form the required geometry. Stereolithography (SL), fused deposition modeling (FDM), polyjet, laminated object manufacturing (LOM), three-dimensional printing (3D printing), laminated engineered net shaping (LENS), selective laser sintering (SLS), and electron beam melting are the most important AM technologies [33]. Out of these techniques, 3D printing has been extensively investigated for the preparation of biocomposites for a variety of applications [[67], [68], [69]]. Considering the applicability of 3D printing in preparing complex biocomposite structures, the work in this area is immense and it is thought to represent the future of biocomposite manufacturing.

## 6 Sliding wear properties of green biocomposites

### 6.1 Sliding wear property of bio-filler incorporated bio-polymer composite

With the growing awareness of the environmental impacts of nonrenewable-derived composites, there has been a rise in interest in the utilization of renewably sourced polymer composites. Biopolymers which are derived from chemicals or renewable sources act as an alternative to conventional synthetic polymers [70]. Biopolymers such as starch, PLA, PGA, and PHB are commonly utilized for making biocomposites for various applications [71]. The significance of biocomposites in low friction and low wear properties, particularly for automotive applications, has been extensively explored as part of the thrust towards sustainable product developments [19]. However, biocomposites using both bio-sourced filler and polymer for wear properties, particularly with a non-fiber natural filler, haven't received much attention. Early studies by Bhuyan et al. showed the influence of bio filler and crosslinking agents on the sliding wear behavior of biocomposites [72]. They fabricated biocomposite using filler as spent germ (a sustainable resource produced as a byproduct of maize grain) in a tung oil-based thermoset polymer resin matrix along with divinylbenzene (DVB) as a crosslinking agent and studied the wear characteristics at the varied composition of both filler and DVB. In the absence of DVB, the composite has shown deteriorated wear characteristics due to the plasticizing effect of filler arised from unreactive crude oil content. However, the addition of DVB at a maximum of 20 wt %, resulted in enhanced polymer-matrix bonding, reduction of coefficient of friction (COF), and wear volume of the composite [72].

Epoxy is one of the major thermoset polymers that have been used for versatile applications due to their high strength, adhesion, resistance to chemicals, thermal resistance, etc. [73] However, considering the global switch towards sustainable development, the replacement of traditional epoxy resins with green bio-based epoxies for various applications has also been studied [74,75]. In addition, the use of bio nanomaterials for multifunctional applications is getting attention due to their high performance and biocompatibility. Barari and colleagues made the first attempt to investigate the tribological properties of nanocellulose reinforced biocomposites [76]. Silylated and non-silylated cellulose nanofiber aerogels were employed as a reinforcement in the bio-epoxy matrix to create the bio nanocomposite. The wear tests were carried out with varying fiber volume, loads (4,7, and 10 N), and siding speeds. Although the effect of sliding speed on nanocomposite wear behavior was insignificant, the application of normal load was found to be having a major role in determining surface wear. At higher loads (10 N), the investigators observed fiber pullouts, large worn volume, and lower COF which was attributed to the plastic deformation and back transfer of polymer materials that occurred when the applied load was beyond the load-bearing capacity of the biocomposite.

**Fig. 4:**
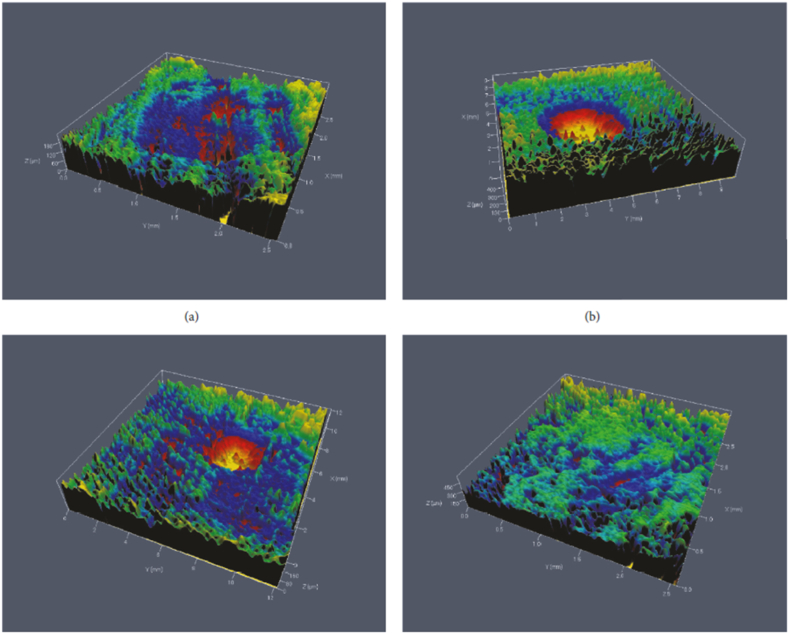
Confocal Laser Scanning Microscopy (CLSM) images for a PLA-biocarbon biocomposite at a) 0 vol %, b) 5 vol %, c) 15 vol %, and d) 30 vol % of biocarbon content [80].

Polylactic acid (PLA), one of the most often used raw polymers in 3D printing, has garnered considerable attention for its biodegradability and eco-friendly characteristics [77]. The incorporation of natural and synthetic reinforcements in PLA for 3D printing applications has been investigated by many researchers [78,79]. However, the attempts to understand the wear behavior of PLA-based biocomposite for 3D printing applications are rarely discussed. Ertane and colleagues described a 3D printed composite for sliding wear experiments that used wheat stem-derived biocarbon reinforced PLA filaments [80]. Four cylindrical-shaped 3D printed composite with a varied composition of biocarbon was tested against an Al_2_O_3_ ball at dry condition under an applied load of 1 N. The investigators observed a significant wear depth, worn volume, and a strong fluctuation in the COF in non-reinforced PLA. As shown in Fig. 4 (a - d), the incorporation of biocarbon, on the other hand, is reported to be impacted by increased stiffness and reduced deformation of PLA composite, resulting in improved wear resistance.

### 6.2 Biopolymer reinforced with synthetic/inorganic filler

Biocomposites consisting of synthetic or non-bio reinforcing agents in biopolymer matrix are still a research hotspot. The innate drawbacks of natural reinforcing fillers such as irregularity in the size, and shape, poor resistance to microbes, moderate thermal stability, difficulty in extraction from natural resources, and high moisture sensitivity & which restricts the widening of their reinforcing applications [81]. As a reinforcement, synthetic fillers provide inherent advantages in biocomposites to address these constraints. Early investigations on the wear properties of biocomposites using inorganic fillers were reported by Bhuyan and colleagues [82]. They fabricated the biocomposite with organo-modified montmorillonite clay (MMT) in a biopolymer matrix derived from food-grade low saturation (LSS) soybean oil. Improved wear characteristics were reported with an optimal clay content of 1 wt % in the composite. However, further incorporation of MMT resulted in agglomeration and embrittlement of the composite. Similar studies were conducted by Iyer and colleagues, where the wear behavior of cellulose acetate phthalate (CAP) and chitosan blend reinforced with functionalized nano clay at varied proportions and different load levels were investigated [83]. The biocomposite with less filler proportion (6 wt %) under a load of 5 N and 10 N demonstrated the best wear property. However, an unstable frictional behavior at both higher loads and filler content was also observed by the investigators, attributed due to the influence of partially brittle polymer blend and possible agglomeration of fillers [83].

Carbon-based reinforcing materials such as carbon fiber, graphite, carbon nanotubes, and graphene have been extensively studied for improving the wear aspects of polymer composites. Despite the self-lubricating property, the lower coefficient of thermal expansion, higher thermal conductivity, and excellent load carrying capacity, enable these materials enticing for developing composites for tribological applications [[84], [85], [86]]. However, the incorporation of carbon-based materials in the biopolymer matrix for wear performances has received far less attention. Deepthi et al. investigated the sliding wear properties of composite by reinforcing functionalized multiwalled carbon nanotube (MWCNT) in chitosan and starch-based thermoplastic matrix [87]. The researchers noticed a significant reduction in wear loss with even 1 wt % of MWCNT incorporated nanocomposite compared to the unmodified polymer at different normal loads up to a value of 26 N. Similarly, Omrani and colleagues fabricated biocomposite through resin transfer molding (RTM) process, using bio-based epoxy matrix derived from wood pulp and bio-fuels, and carbon fibers reinforcement [88]. They focused on the impact of fiber volume fraction (up to 30 vol %), sliding speeds (up to 0.35 m/s), and normal load (up to 20 N), in sliding wear behavior and COF of the composite, and yielded a few significant observations as follows.

**Fig. 5:**
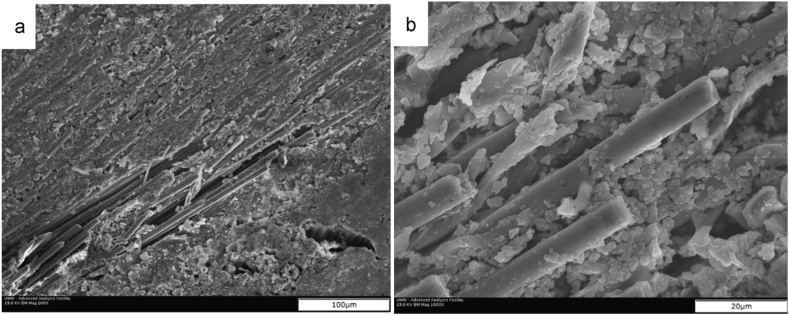
SEM images of fiber-pullouts at a) 200 ×, b) 1000× magnifications for 30 wt % carbon fiber reinforced bio-epoxy composite at 20 N normal load and sliding speed of 0.15 m/s [88].

a) As the fiber volume fraction in composite increases, the COF and wear loss decrease.
b) Value of COF was insignificant at varied sliding speeds, while the least volume loss was observed at lower normal loads and higher fiber volume fractions for every sliding speed.
c) At higher normal loads, there was a decrease in COF and increase in wear loss values, which was attributed to a large number of fiber pullouts [Fig. 5 (a, d)] causing a lubrication effect as well as an increase in surface roughness of the composite.

Attempts to produce polymer composites using 3D printing techniques, particularly for developing products that are subjected to wear, are less explored. As aforementioned, given the inherent benefits of carbon-based materials, efforts were made to fabricate PLA-graphite biocomposite via 3D printing [89]. With the inclusion of 1 wt % of graphite content into the PLA matrix, the investigators observed an improvement of reduction in wear loss and COF of biocomposite compared to non-reinforced PLA. Further addition of graphite up to a 10 wt % bought a 65% reduction in wear rate, which was attributed to graphite film formation on the sliding contact surface. It is worth mentioning that, along with the improvement in wear behavior, the composite was reported to be more rigid with the inclusion of graphite, resulting in a decrease in strength and impact strength [89]. However, considering the application as sliding elements where the polymeric parts are loaded with less force, the modest drop in strength is inconsequential in practice. Similarly, efforts to fabricate polylactic acid-polyether ketone ketone-hydroxyapatite-chitosan (PLA-PEKK-HAp-CS) biocomposite through the 3D printing technique have also been reported [90]. The investigators observed a 25% reduction in wear loss in prepared composite compared to neat PLA. However, these attempts pave the way for the fabrication of sustainable 3-D printed parts for wear applications in a wider perspective.

**Fig. 6:**
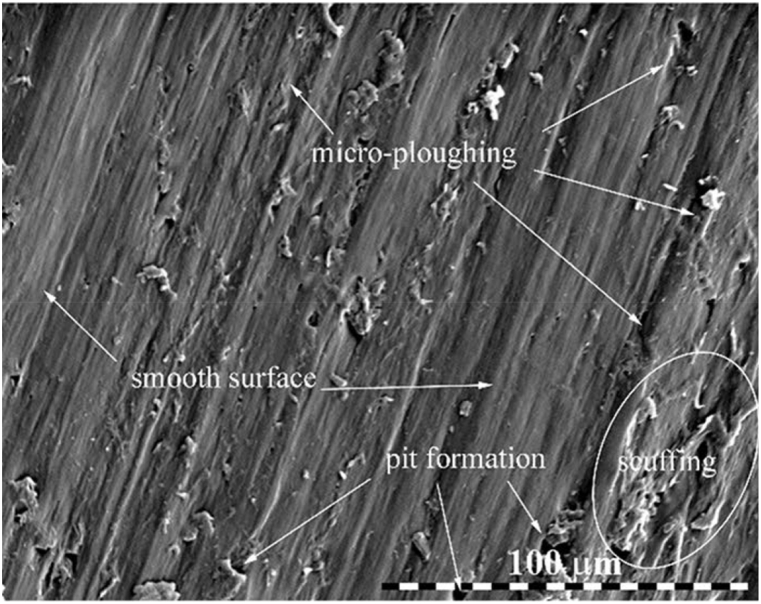
SEM image of marble dust-PLA biocomposite surface at higher sliding wear testing parameters [96].

Apart from carbon-based materials, MoS_2_ and PTFE as a reinforcement in the composite have demonstrated their self-lubricating properties for wear application [[91], [92], [93]]. Considering the low frictional coefficient, lubrication, and biocompatibility [94] of MoS_2_, investigations have been carried out to comprehend the wear properties of MoS_2_ reinforced hydroxypropyl methylcellulose (HPMC) biocomposite [95]. The investigators observed a reduction in COF of 70% even at 1 wt % of MoS_2_, attributed to the transfer layer formation contact surface of composite, due to the delamination of MoS_2_. Recently, Lendvai and coworkers prepared biocomposite using waste marble dust in PLA via melt blending [96]. Upon examining the various combination of key parameters in the sliding wear test of composite, it was observed that the normal load, followed by sliding velocity, has a considerable effect in determining the wear behavior of composite. Keeping the conditions as 20 wt % of filler concentration, lower sliding speed, and normal load the composite exhibited enhanced sliding wear resistance. On the other hand, irregularities such as scuffing, micro-ploughing, peeling-off, and pit formation (Fig. 6) on the composite surface were observed with a higher normal load and sliding speed testing condition. Furthermore, the authors concluded that, with a combination of lower normal load, sliding distance, sliding velocity, and higher filler content, the biocomposite can achieve the optimal wear properties [96]. However, the reinforcement of marble dust in PLA improves the composite property whilst mitigating the environmental issues assisted with disposed waste marble dust.

### 6.3 Bio filler synthetic polymer composite

Bio sourced fillers other than natural fibers have piqued the interest of researchers over the past decade, to obtain biocomposites featuring desired sliding wear properties that could replace traditional alternatives. In certain applications where components are subjected to weathering, temperature, heavy load, and other external factors, a complete replacement of both filler and polymer from completely bio-origin are not recommended. In such circumstances, synthetic polymers such as epoxy, polypropylene, polyesters, polyethylene, and polyvinyl chloride, are typically utilized according to the application requirements. Nevertheless, because of the economic benefits of lower material cost, low density, and abundant raw material availability, fillers from agricultural waste, bi-product of wood processes, and animal wastes are considered widely as a reinforcement for the preparation of biocomposites [97].

#### 6.3.1 Agro waste fillers in reinforced biocomposite

Generally, agricultural wastes are left to nature, burned, or used as a fuel source for household purposes. Improper disposal, such as burning agricultural waste, does have a harmful influence on the environment. Sustainable techniques to solve this problem, such as employing agricultural waste as a reinforcement for polymer matrix to create eco-friendly polymer composites, have been extensively investigated over the last few decades [98]. When it comes to tribological qualities, composite materials comprising agricultural residues as fillers have received a lot of attention [99]. However, the influence of these additives on the sliding wear properties of composites has received scant attention.

In countries like India, rice is among the widely cultivated agricultural crop. Rice husk, an agricultural waste that is the outer layer of paddy grains, is used as a promising filler for polymer composites. Studies have been framed to understand the mechanical and physical properties of rice husk-filled polymer composites [100]. However, there are very few papers that demonstrate the effect of rice husk on the sliding wear behavior of polymer composite. Rout and Satapathy fabricated rice husk-filled epoxy composite for analyzing the dry sliding behavior of the composite [101]. They observed that the sliding wear behavior is influenced by the testing parameters such as filler content, sliding speed, and normal load. It was reported that the impact of rice husk on minimizing wear loss from the sliding surface of composite was considerable at a filler level of 20 wt %. Similar studies utilizing hybrid fillers in epoxy matrix indicated that the effect of rice husk filler content had the greatest importance on composite wear behavior, followed by other wear parameters such as applied load and sliding speed [102]. It is worth noting that, poor filler impregnation can have a detrimental impact on the composite's tribological and mechanical characteristics. Methods like Surface treatment of the filler with chemicals or plasma treatments, coupling agents, compatibilizer, and electron beam irradiation have been investigated for better filler homogeneity in the composite [103,104]. Apart from rice husk, agricultural waste materials like wheat husk also act as polymer reinforcements. In studies comparing the sliding wear behavior of wheat husk, coir fibers, and rice husk reinforced polymer composites, the rice husk-based composites had shown an inferior wear resistance and low COF values over the other two fillers [105].

Bagasse ash is another agro-waste filler with good wear-resistant qualities. In polymer composites, it prevents matrix component fragmentation by limiting sliding subsurface contact and also generates a transfer layer on the sliding surface, resulting in enhanced wear resistance in the composite. Moreover, the reinforcement of bagasse ash increases hardness and lowers the coefficient of thermal expansion of the polymer matrix, leading to enhanced thermal stability and mechanical properties of the composites [106]. Atunya et al. investigated the wear behavior of breadfruit seed husk ash (BFSHA) incorporated with recycled polyethylene biocomposite [107]. Even though the integration of BFSHA filler showed a good impact on the wear resistance, it was observed that the sliding speed and applied load had a stronger influence on the enhanced wear behavior of the composite [107].

Date palm seed, as a reinforcing filler, on their mechanical, chemical, and physical properties with polymer composites has also been well researched [[108], [109], [110]]. However, the effect of date palm seed on the tribological properties of polymer composites has received very little attention. Ibrahim examined the sliding wear behavior of date seed powder/polyester biocomposite using the pin-on disc technique [111]. At a medium sliding speed and normal load, a 25 wt % filler content led to a considerable reduction of COF and wear rate of the prepared composite. On the other hand, with an increase in filler content beyond 15 wt %, elevated normal load, and sliding speed, the wear resistance of the composite was found to be declined [111]. The same author also facilitated similar investigations employing palm fronds and mango dry leaves in polyester resin to further broaden the scope of agro-waste fillers in sliding wear applications [112]. Upon examining the wear properties of both the fillers, the findings indicated that the palm frond/polyester composite is best suited for applications such as brake pads owing to high friction and low wear rate.

While dry mango leaves/polyester composite were recommended for applications such as solid lubricant given the low friction coefficient and high wear resistance [112]. Furthermore, various agro wastes such as Jatropha seed cake particulate, walnut shell powder, pongamia pinnata seed, coconut shell powder, almond shell powder, and corn cob ash, are being studied for the sliding wear behavior of polymer composites [[113], [114], [115], [116], [117], [118], [119]].

#### 6.3.2 Wood-based filler reinforced biocomposites

Wood flour, as a naturally sourced reinforcing agent, has been widely used in the fabrication of wood polymer composite laminates. The peculiarities such as greater strength to weight ratio, easy processability with polymer matrix, and renewability give much significance to wood-based fillers for utilization in polymer composites. In addition, wood powder included composites have conferred thermal insulation qualities, which can minimize heat generation owing to friction and further decrease of wear. Early studies on wood flour epoxy composite, comparing the sliding and abrasive wear behavior were conducted by Dwivedi and colleagues [120]. Beyond a filler level of 20 wt %, the investigators observed a reduction in the sliding wear resistance of the composite. While abrasive wear resistance was enhanced above a filler content of 30 wt %. Kranthi and Satapathy investigated the influence of pinewood powder on sliding wear characteristics using an epoxy matrix [121]. With varied filler contents in the epoxy matrix, the investigators found that pinewood powder improves the sliding wear resistance of the prepared biocomposite. Similar studies were performed by Karthik et al. to evaluate the effect of different content (0, 5, and 10%) of pine wood dust powder with epoxy matrix [122]. The incorporation of pine wood dust powder was found to be enhancing the wear resistance of prepared composites. Mishra examined the sliding wear behavior of teak wood dust reinforced epoxy composite using three different sized wood dust: 150, 212, and 300 μm [123]. Out of the three prepared composites, it has been reported that the composite with 150 μm sized wood dust has shown enhanced wear resistance properties along with other mechanical performances.

The incorporation of wood flour as a reinforcing filler increases the hardness and the frictional coefficient of thermoplastic polymer composites. However, it should be emphasized that the addition also increases water absorption, causing deterioration of mechanical properties of the composites [124]. Kumar et al. added varied proportions of mango, sheesham, mahogany, and babool dust to the polypropylene matrix to improve the mechanical as well as wear resistance of the composite [125]. It was established that, increasing the filler content impacted in reduction of wear resistance for all kinds of prepared composites. Furthermore, the composites made of babool and sheesham dust were reported to have higher mechanical qualities, whilst the composites made of mango and mahogany reinforcement were found to be more apparent be wear-resistant [125].

Wood-derived fillers, with an abundance of hydroxyl groups in their structure, are inherently hydrophilic, whereas polymers are generally hydrophobic. Incorporation of hydrophilic filler in hydrophobic polymer usually results in weak interfacial interaction and filler agglomeration, restricting the mechanical properties of the composites [126]. To overcome these challenges, and to achieve even distribution and good dispersion of fillers in the polymer matrix, methods such as the addition of crosslinking agents and binders, modification of fillers and polymers, and gamma radiations, are extensively applied [127,128]. Ibrahim et al. used maleic anhydride-modified polypropylene (MA-PP) matrix for preparing the biocomposites for sliding wear applications [129]. With hydrophilic wood flour, it is obvious to expect a good interfacial interaction of polar groups of maleic anhydride chains to enable enhanced properties. The investigators discovered improved wear resistance properties for the composite, which was consistent with the expected filler-polymer interaction.

#### 6.3.3 Animal sourced fillers reinforced biocomposite

Bio fillers derived from animal source have been widely researched for fabricating polymer biocomposites. Animal fibers such as wool, silkworm silk, and spider-silk have demonstrated their ability as a reinforcing agent in a range of areas involving, fire-resistant, electronic, aerospace, and biomedical applications [[130], [131], [132], [133]]. Animal wastes, such as chicken feathers, bones of animals, and shells of sea organisms, are another set of biomaterials, which can be used as an alternative reinforcement for polymer composites [134]. In developing countries, where proper disposal of animal wastes is still a challenge, exploring the potential of these biomaterials can pave the way to the development of novel materials for polymer reinforcement as well as solve waste management problems. Taking this all into consideration, attempts have been made to develop polymer composites with bone ash for enhancing the mechanical properties of polymer composites [135]. Researchers had investigated the potential of animal waste, particularly cow bone, as a reinforcing filler in recycled polyethylene matrix for sliding wear applications [136]. The integration of bone particles, as a hard material, provided improved load-bearing capacity, subsequently improving the mechanical characteristics and wear resistance of the prepared bio-composite [136]. Another naturally available hard material, periwinkle (sea snail) shell, owing to its higher calcium content, has also been explored to assess the impact on wear properties of polymer composite [137]. As a hybrid filler in polyester resin, at 5 wt % proportion, the periwinkle shell has shown improved wear properties in the composite. Recently, gaint African snail (lissachatina fulica) has also been explored for their sliding wear behavior for developing automotive brake pads [138]. Table 1 summarizes the parameters of biocomposites in tribological testing.

**Table 1:** Tabular presentation of all the parameters of biocomposites in tribological testing.

Polymer	Filler	Fabrication method	Normal load	Material of contact surface	Sliding speed	Sliding distance	Filler content	COF	Type of test	Wear Loss/wear depth	Ref.
**Bio-filler incorporated bio-polymer composite**											
Tung oil derived thermoset resin	Spent germ	Compression molding followed by oven curing	800 mN	diamond	5 mm/s	30 *m*	40 wt %	0.24	Ball on flat	2.4 μm (wear depth)	[72]
Tung oil derived thermoset resin	Spent germ	Compression molding followed by oven curing	15 N	alumina	0.123 m/s	200 *m*	40 wt %	–	Pin on disk	0.02 g (mass loss)	[72]
Bio Based epoxy (Super-Sap Entropy®)	Cellulose nano-fibers (Silyated)	Liquid Composite Molding (LCM) pro-cess	10 N	stainless steel	0.15 m/s	1 km	1.4%	0.35	pin-on-disk	2 *10^−7^ mm^3^ (volume loss)	[76]
Polylactide (PLA)	Biocarbon	3 D printing	1 N	Alumina	–	–	30 wt %	0.5	translatory oscillation	0.1 mm^3^ (volume loss)	[80]
**Biopolymer reinforced with synthetic/inorganic filler**											
Conjugated low saturated soybean oil with styrene-based polymer	Montmorillonite clay	Molding	500 mN	Steel	5 mm/s	60 *m*	1 wt %	0.3	ball-on-flat	2.33 μm (wear depth)	[82]
Cellulose acetate Phthalate and Chitosan blend	Silane treated nanoclay	Compression molding	5 N	EN31 Alloy Steel	60 rpm	950 *m*	6%	–	pin-on-disk	60 μm (wear depth)	[83]
Chitosan and starch blend	multiwalled carbon nanotube	Compression molding	6 N	–	1.2 m/s.	–	1 wt %	–	pin-on-disk	0.05 g (mass loss)	[87]
Bio-based epoxy (Super-Sap Entropy®)	Carbon fiber	resin transfer molding	20 N	stainless steel	0.25 m/s	1000 *m*	30 wt %	0.12	pin-on-disk	1 *10^−7^ mm^3^ of volume loss (at 10 N normal load)	[88]
Polylactide	Graphite	3 D printing	5.49 N	Steel	0.34 m/s	3000 *m*	1 wt %	0.4	pin on disc	11*10^−3^ μm/m of wear rate (at 10 wt % of graphite)	[89]
polylactic acid-polyether ketone	hydroxyapatite-chitosan	extrusion	1 kg	–	277 r/min.	10.8 *m*	25 wt %	0.057	pin-on-disc	316 μm (wear depth)	[90]
Hydroxypropyl methylcellulose	MoS_2_	Casting	2 N	chrome steel	0.03 m/s.	–	5 wt %	0.15 (at 1 wt %)	ball-on-disk	60 * 10^−3^ mm^3^ (volume loss)	[95]
Poly (Lactic Acid	Waste Marble Dust	Extrusion followed by injection molding	10 N	–	1 m/s	400 *m*	20 wt %	–	pin-on-disc	11.4 * 10^−3^ mm^3^ (volume loss)	[96]
**Synthetic polymer reinforced with bio fillers**											
Polyethylene	Breadfruit seed hull ash particulate	Compression molding	30 N	Steel	3 m/s	5000 *m*.	25 wt %	0.3	pin-on-disc	5.03 * 10^−4^ g/m (wear rate)	[107]
Polyester	Date seed powder	Casting	0.636 MPa	–	4 m/s	–	10 wt %	0.8	pin-on-disk	–	[111]
			0.318 MPa	–	3.5 m/s	–	25 wt %	–		6.6 * 10^−4^ g/m (wear rate)	
Polyester	Palm fronds	Casting	6 N	Steel	0.8 m/s	–	50 wt %	0.8	pin-on-disk	1 * 10^−4^ g/m (wear rate)	[112]
Polyester	Mango leaves	Casting	6 N	Steel	0.8 m/s	–	50 wt %	0.42	pin-on-disk	1.8 * 10^−4^ g/m (wear rate)	[112]
Epoxy	Jatropha Curcas L. seeds	Casting	20 N	Steel	–	300 *m*	20 wt %	0.25	ball-on-flat	7 * 10^−5^ g (mass loss)	[139]
Polypropylene	Olive Pit	Casting	5 N	–	100 mm/min	40 *m*	10 wt %	0.9	Pin-on-disc	0.1 mm^3^/Nm (wear rate)	[119]
Epoxy	jatropha seed cake waste	Open resin molding	5 N	–	0.5 m/s	500 *m*	40 wt %	0.23	Pin-on-disc	2 * 10^−7^ g/Nm (wear rate)	[117]
Epoxy	Wood flour	Casting	80 N	Stainless steel	2.2 m/s	8000 *m*	20 wt %	–	Pin-on-disc	5 *10^−15^ m^3^/Nm (wear rate)	[120]
Epoxy	Teak wood dust	Casting	30 N	steel	1.5 m/s	5000 *m*	10 wt %	0.64	Pin-on-disc	100 μm (wear loss)	[123]
Polypropylene	Babool	Injection molding	–	–	–	–	20 wt %	–	Pin-on-disc	6 g (mass loss)	[125]
Polypropylene	Mango wood		–	–	–	–		–		5.5 g (mass loss)	
Polypropylene	Sheeshum		–	–	–	–		–		5.5 g (mass loss)	
Polypropylene	Mahagony		–	–	–	–		–		6.5 g (mass loss)	
Maleated Polypropylene	Wood flour	Injection molding	10 N	Steel	20 mm/s	–	50 wt %	–	Ball on disc	1*10^−5^ g (mass loss)	[129]
Polyethylene	Cow bone powder	Compression molding	10 N	Steel	1.18 m/s	70.8 *m*	25 wt %	–	pin-on-disc	27 *10^−4^ g (mass loss)	[136]
Polyester	charcoal particles	Casting	10 N	Steel	200 rpm	4.3 *m*	20 wt %	–	Pin-on-Disc	0.015 mm^3^/Nm (wear rate)	[137]
Polyester	charcoal particles and periwinkle shell particles hybrid (75:25 wt ratio)	Casting	10 N	Steel	200 rpm	1.5	5 wt %	–	Pin-on-Disc	0.013 mm^3^/Nm (wear rate)	[137]

## 7 Conclusion

Sliding wear is responsible for three main parameters i.e., the adherence of material, damage of surface asperities under load, and the fracture of material in the substrate. These deformations will affect the loss of materials from the substrate. Several techniques are followed to control the behavior of the materials under sliding friction. In this literature review, the authors try to discuss several methods to achieve better performance of biocomposites. For instance, sliding wear property of bio-filler incorporated bio-polymer composite, biopolymer reinforced with synthetic/inorganic filler and finally bio filler synthetic polymer composite that comprises of agro-waste fillers in reinforced biocomposite, wood-based filler reinforced biocomposites and animal-sourced fillers reinforced biocomposites.

## Author contribution statement

All authors listed have significantly contributed to the development and the writing of this article.

## Funding statement

This research was funded by National Science, Research and Innovation Fund (NSRF), and King Mongkut's University of Technology North Bangkok with Contract no. KMUTNB–FF–66-14.

## Data availability statement

No data was used for the research described in the article.

## Declaration of interest's statement

The authors declare the following conflict of interests: The paper's corresponding author, Sanjay Mavinkere Rangappa, works as an Associate Editor for Heliyon Materials Science.
